# The Role of Oxidative Stress in the Aging Eye

**DOI:** 10.3390/life13030837

**Published:** 2023-03-20

**Authors:** Deniz Goodman, Steven Ness

**Affiliations:** 1 Boston University Chobanian & Avedisian School of Medicine, Boston, MA 02118, USA; 2 Department of Ophthalmology, Boston Medical Center, Boston, MA 02118, USA

**Keywords:** oxidative stress, reactive oxygen species, eye, age, age-related

## Abstract

Given the expanding elderly population in the United States and the world, it is important to understand the processes underlying both natural and pathological age-related changes in the eye. Both the anterior and posterior segment of the eye undergo changes in biological, chemical, and physical properties driven by oxidative stress. With advancing age, changes in the anterior segment include dermatochalasis, blepharoptosis, thickening of the sclera, loss of corneal endothelial cells, and stiffening of the lens. Changes in the posterior segment include lowered viscoelasticity of the vitreous body, photoreceptor cell loss, and drusen deposition at the macula and fovea. Age-related ocular pathologies including glaucoma, cataracts, and age-related macular degeneration are largely mediated by oxidative stress. The prevalence of these diseases is expected to increase in the coming years, highlighting the need to develop new therapies that address oxidative stress and slow the progression of age-related pathologies.

## 1. Introduction

According to the U.S. Census Bureau, nearly a quarter of Americans will be older than 65 years of age by 2030, and the number of Americans 85 years of age and older will triple by 2060 [1]. In fact, the federal agency projects that by 2060, the elderly will outnumber children for the first time in U.S. history [1]. This expansion of the elderly population underscores the need for an understanding of the changes in the human body that occur as a result of the natural aging process.

Several of the most salient changes are vision-related, and the elderly often experience difficulties in their ability to accommodate to light, discriminate between colors, and view close objects [2]. The prevalence of eye conditions involving the eyelids, extraocular muscles, cornea, lens, and retina increases with age (Table 1) [3,4,5]. Given that vision impairment has been associated with problems performing activities of daily living and with psychiatric conditions [6,7], it is important to understand the changes that occur in the eye as a result of growing older. We present a review of the literature regarding the normal changes in ocular anatomy and function that are secondary to aging (Table 2 and Table 3). Our work discusses the role of oxidative stress in the pathophysiology of the three most common ocular pathologies that occur with aging, including cataracts, glaucoma, and macular degeneration.

## 2. External and Anterior Segment Changes

### 2.1. Eyelids and Lacrimal Glands

Aging in the face, including the eyelids, is driven by a lower regenerative capacity and upregulated degradative enzyme activity [8]. These changes cause collagen degradation, especially in the deep dermis. As aging continues, extracellular matrix components lose their interwoven organization and tight interactions [9].

Beyond tissue atrophy, the lower eyelid often undergoes horizontal lid laxity [4,8]. The anterior inferior periocular soft tissue and orbital fat pad expand in volume with aging [8,10], leading to a “sunken in” appearance to the eyes and pronouncing lid laxity. Continued lid laxity leads to ectropion, or eversion of the eyelid margin. However, if the pretarsal orbicularis muscle maintains its strength while the midface sags during the aging process, one instead experiences entropion, or inversion of the lower eyelid [4]. Either of these conditions can result in foreign body sensation, tearing, or corneal damage [8].

The upper eyelids can be impacted by dermatochalasis and blepharoptosis. Dermatochalasis is the product of external environmental insults and redundant upper eyelid skin [8]. Blepharoptosis, on the other hand, results from loss of muscle tone or dehiscence of the levator palpebrae superioris muscle and the Müller muscle [8,11]. Flament et al. compared digital photographs of women from six different ethnicities and found that as age progresses, the height of the eyes decreases due to the sagging of the upper eyelid and the angle between the eye length and the horizontal inter-pupillar line decreases from the sagging of the outer corner of the eyes [12]. 

Dry eye is common among the elderly due to age-related changes in the lacrimal and meibomian glands. Histopathologic changes in lacrimal glands include atrophy, fibrosis, ductal dilation, and proliferation, as well as lymphocytic and fatty infiltration [13,14,15,16,17]. Similarly, meibomian glands undergo atrophy [13,15]. The pathogenesis of dry eye also involves oxidative stress, as the lacrimal glands of aged mice showed an accumulation of 8-hydroxydeoxyguanosine and lipofuscin-like inclusions, among other oxidative stress biomarkers [18]. Over time, the ocular surface and tear film become compromised [18]. These changes manifest as diminished tear production, resulting in a loss of contrast sensitivity and functional visual acuity, as well as dry eye symptoms including burning, epiphora, and foreign body sensation [13,14,15,16,17].

### 2.2. Sclera

There exists conflicting data on the association between changes in the thickness of the sclera and age [19,20,21,22]. However, an increase in the stiffness and rigidity of the sclera is well established and has been attributed to a decrease in connective tissue and extracellular matrix components, including diminution of elastin fibers, decorin, and biglycan [23,24]. Additionally, the sclera stiffens with age due to elevated fibril crosslinks and tropocollagen molecules per fibril [23]. The formation of senile scleral plaques (calcium sulfate or calcium phosphate at the insertions of medial and lateral rectus muscles) may also contribute to increased scleral rigidity in older patients [25,26]. Individuals of African descent appear to experience a faster rate of increase in age-related rigidity compared to individuals of European descent, which may be linked to greater glaucoma prevalence in elderly African Americans [27].

### 2.3. Cornea

**Figure 1:**
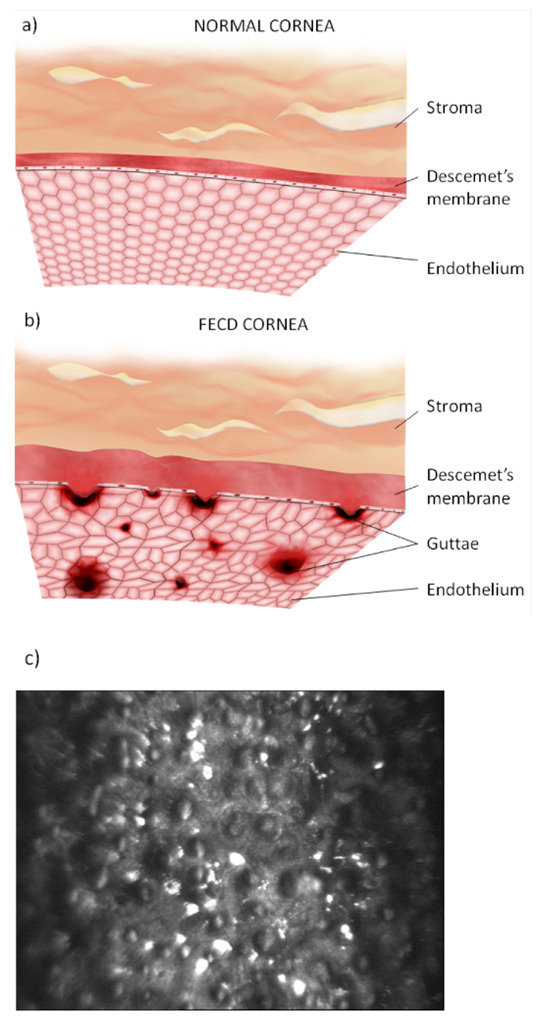
Endothelium and Descemet’s membranes from normal cornea (**a**) and cornea in Fuchs’ endothelial corneal dystrophy (FECD) (**b**) showing structural changes, including guttae formation, modification of the hexagonal endothelial cell mosaic and Descemet’s membrane thickening in FECD. A representative confocal microscopy image of endothelial guttae in an advanced stage of FECD obtained from a patient of the Department of Ophthalmology, Medical University of Warsaw, Warsaw, Poland is shown in part (**c**) [29].

The aging cornea undergoes alterations in both optical and physical properties, degeneration, and a reduction in immunological capacity [4,26]. Histologic changes in the cornea include thickening of the Descemet’s and epithelial basement membranes, in addition to decreases in the corneal stromal cell density as well as corneal endothelial cell and conjunctival keratocyte numbers [28]. Fuchs’ dystrophy is an age-related loss of corneal endothelial cells accompanied by the deposition of extracellular material (guttae) on Descemet’s membrane (Figure 1) [28,29]. The decline in corneal endothelial cells leads to hypertrophy in the remaining endothelial cells as a compensatory response [4,28]. As endothelial cell counts fall below critical levels, they lose the ability to adequately dehydrate the cornea, resulting in edema and lower corneal clarity [28].

Previous studies have demonstrated an increased prevalence of astigmatism with advancing age and a shift from with-the-rule to against-the-rule astigmatism [4,30,31]. Against-the-rule astigmatism, characterized by a steeper horizontal corneal curvature, results from altered mechanical properties of the eyelid, corneal stroma, Descemet’s membrane, and extraocular muscles [30].

Corneal degenerative changes are visibly evident throughout the corneal layers [4]. The deposition of white-grey lipid opacities in the peripheral cornea is termed arcus senilis [32]. Cornea farinata is identified throughout the corneal stroma by its small, grey, and opaque appearance [33]. Central and posterior corneal stroma may have crocodile shagreen, which is characterized by classic polygonal opacities akin to crocodile skin [34]. While each of these conditions are usually asymptomatic, their incidence rates are directly proportional to age.

The cornea also becomes immunocompromised with age. Animal studies have shown phagocytic dysfunction and a loss of phagocytically active cells in the corneas of older mice, resulting in impaired recovery from gram-negative bacterial infections [35]. Older mice were also found to have a defective response to Pseudomonas infection due to decreased upregulation of intercellular adhesion molecule 1, delaying infiltration of polymorphonuclear cells into the cornea and allowing for unchecked bacterial growth [36]. These immunological changes found in mouse models may have implications for the human population. Infectious keratitis presents more severely in the elderly population compared to the younger population [37]. Constantinou et al. found that the majority of corneal ulcers among the elderly are secondary to non-healing microbial keratitis caused by *Pseudomonas aeruginosa* [38].

### 2.4. Trabecular Meshwork

The trabecular meshwork (TM) undergoes changes in shape, cellularity, and pigmentation as a function of age. A reduction in the TM height leads to the meshwork assuming a rhomboid shape in older eyes [26,39,40]. The cellularity of the TM declines linearly with a loss of 0.58% of cells per year and a 47% decline in absolute cell number over an 81-year study period [41]. The number of TM stem cells was also found to diminish over time [42]. These changes, in conjunction with an increase in extracellular components and the narrowing of aqueous humor outflow tracts, are responsible for greater resistance to aqueous humor outflow with age [43]. Elevated intraocular pressure, resulting in part from obstructed aqueous outflow, accounts for the higher prevalence of glaucoma in older patients. Lastly, prior studies have reported TM hyperpigmentation due to increased melanin in older patients [4,44].

### 2.5. Ciliary Body

The ciliary body is subject to changes in the morphology, cellularity, and collagen content of its stroma [26]. The muscle has a tendency to shorten and widen while its internal apical edge moves forward over time [45]. The nasal and temporal maximum ciliary muscle thickness increases with age [46]. Additionally, the diameter of the unaccommodated ciliary muscle decreases during the aging process [47]. Histologically, the ciliary body loses vascularization and cellularity while accumulating collagen [26].

### 2.6. Crystalline Lens

The lens undergoes age-dependent changes in geometric parameters. The radii of curvature, lens volume, surface area, cross-sectional area, diameter, lens thickness, and weight linearly increase as a function of time [46,48].

**Figure 2:**
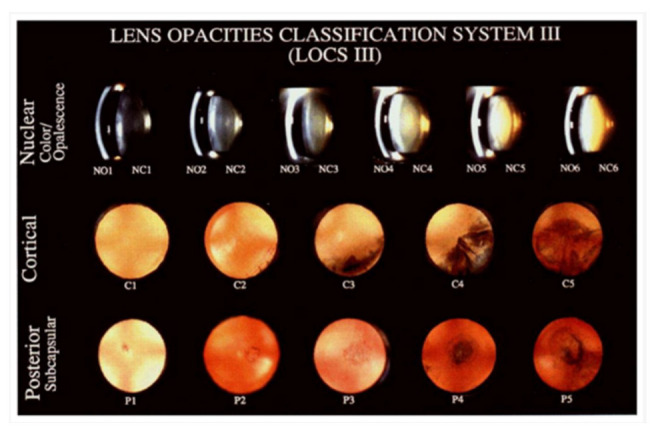
Cataract progression as measured by the Lens Opacities Classification System (LOCS) III grading system [51].

The elderly may have “blue blindness” from the yellowing of the aging crystalline lens [4]. This yellowing increases the absorption of light with shorter wavelengths, including blue light, impairing circadian photoreception [49,50]. Given that blue light is partially responsible for melatonin suppression, the lowered blue light transmittance can lead to mental health disorders in the elderly, including insomnia and depression [50]. Nuclear sclerotic cataracts, posterior subcapsular cataracts, and Morgagnian cataracts arise from age-dependent crystalline lens changes (Figure 2) [26,51]. Cataractogenesis is driven by elevated oxidative stress and the accumulation of oxidized lens proteins [52]. Age-related cataract formation is discussed in greater detail in Section 4.3 of this paper.

Changes in both the ciliary body and lens result in presbyopia, the natural loss of accommodative ability with age. While the contractility and the ring diameter of the ciliary body are unaffected by age [45,53], there is an age-linked reduction in forward ciliary body movement, which is compensated for by greater centripetal ciliary movement [54,55]. Lenticular changes contributing to presbyopia include a 450-fold elevation in stiffness, particularly in the nucleus [56,57]. Biochemical shifts underlying this increased stiffness include an accumulation of cholesterol, dihydrosphingomyelin, and sphingomyelin, as well as a decline in phosphatidylcholines in lens fiber cell membranes and α-crystallin proteins in lens fiber cells. These changes collectively result in decreased membrane and lens flexibility [57,58].

## 3. Posterior Segment Aging Changes

### 3.1. Vitreous Humor

Age-related changes to the vitreous humor are the result of structural changes to its component collagen fibrils and hyaluronic acid [4]. Aggregates of type II collagen occupy fluid-filled lacunae and promote liquefaction of the vitreous body [59,60]. With increasing age, these collagenous fibrils gain motility [59]. When over half of the vitreous body has undergone liquefaction, the posterior vitreous cortex detaches from the retina in a condition known as posterior vitreous detachment (PVD), which may be complicated by a retinal tear from vitreoretinal adhesions or anterior contraction of the vitreous body in up to 14% of cases [4,26,59,61]. Other complications of PVD include intraocular hemorrhage, rhegmatogenous retinal detachment, an epiretinal membrane, and macular holes [62,63].

The vitreous body experiences other structural and physical changes as well. The vitreous base undergoes thickening, particularly in its temporal and posterior portions, and the resulting vitreoretinal traction is involved in the development of retinal tears and rhegmatogenous retinal detachment [61,64]. Stiffness, dehydration, and mobility of the vitreous humor increase as a function of age, while viscoelasticity decreases as liquefaction progresses [63,65,66].

### 3.2. Retina and Retinal Pigment Epithelium

Aging in the retina is characterized by neuronal cell degradation, morphological changes in the retinal layers, and retinal vascular changes. Neuronal cell loss involves a depletion of retinal ganglion cells and photoreceptors, with a greater decline in rods than cones [4,26,67,68,69]. Animal studies have found a shorter outer segment length of rod photoreceptors, but no age-related changes in disc density [70]. There is also an age-linked reduction in the density of rod bipolar cells, which synapse with rod photoreceptor cells [71].

There are several age-dependent microvascular changes as well, especially in disease states. The total retinal blood vessel area, as well as the number of vessels, bifurcation points, and termination points diminish with age [72,73]. The basement membranes of the retinal capillaries undergo thickening and vacuolization, processes that are accelerated by the presence of diabetes [74]. While aging decreases both the number of supportive pericytes and endothelial cells, diabetes only leads to a loss of pericytes [74].

The retinal pigment epithelium (RPE) rests atop Bruch’s membrane and interacts with the vascular choriocapillaris and photoreceptor cells for nutrient and waste exchange. RPE cells outside of the macula increase in width and decrease in height with advancing age [26,75], leading to a generalized thinning of the peripheral RPE layer. The cytoplasm of RPE cells decreases in volume [4], and the cells become progressively vacuolated and pleiomorphic with respect to size, shape, nuclei, and pigmentation [4,75]. Melanin and lipofuscin, two types of pigment molecules found within RPE cells, also undergo age-related changes [76]. There is a tendency for lipofuscin to accumulate with age, and the main fluorophore of lipofuscin, A2E, causes oxidative stress and damages membrane-bound organelles, potentially injuring RPE cells [26,76,77,78]. Lipofuscin also suppresses the antioxidant capacity of melanin [76,79]. Melanin antioxidant properties also diminish due to age-specific photobleaching of melanosomes [76,80]. Lastly, melanin pigmentation of the peripheral retina declines in older patients [4,75,76].

### 3.3. Choroid

Although changes in the choroidal vascularity index and stroma-to-vessel volume are age-independent, the mean thickness, vessel volume, and stroma volume are lower in the elderly population [81,82]. Furthermore, the choriocapillaris density and diameter diminish as a function of age, and this process is pronounced in patients with early age-related macular degeneration (AMD) [26,83]. Choroidal melanocytes show an age-related fusion of melanosomes into rosettes, an accumulation of irregular lipofuscin granules that bind to melanosomes, and an overall loss of melanosomes [84].

### 3.4. Macula and Fovea

**Figure 3:**
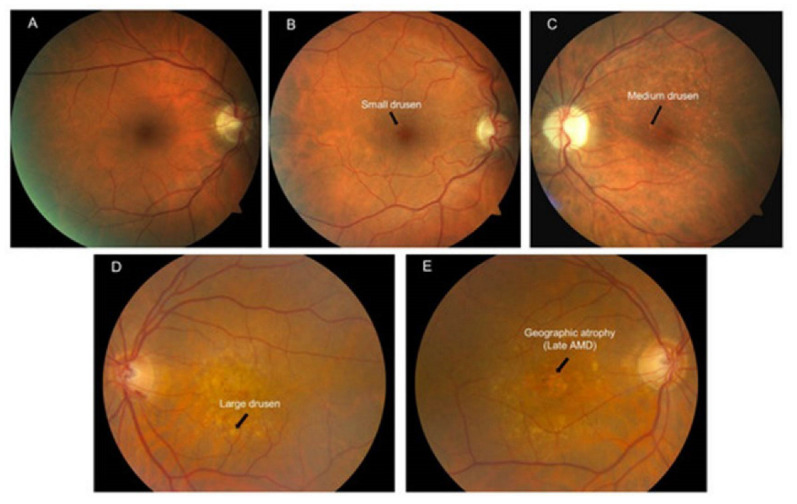
Illustration of the Beckman’s clinical classification of AMD based on color fundus photos. (**A**) No apparent aging changes; (**B**) Normal aging changes; (**C**) Early AMD; (**D**) Intermediate AMD; (**E**) Late AMD as evidenced by geographic atrophy [89].

The macula undergoes anatomical changes secondary to oxidative damage that may predispose elderly patients to AMD [85]. In contrast to peripheral RPE cells, macular RPE cells increase in height over time [26]. Furthermore, Bruch’s membrane thickens and calcifies, which impairs the diffusion of amino acids and other macromolecules [26,83,85,86,87]. Debris from the metabolic turnover of RPE cells, which is mainly composed of lipofuscin and fatty acids, collects on Bruch’s membrane. Subretinal deposits known as drusen appear as yellowish-white opacities between the basal lamina of the RPE and the inner collagenous layer of Bruch’s membrane [4,88]. Age-dependent drusen are the hallmark of AMD, which will be discussed in Section 4.4 of this review (Figure 3) [89]. In contrast to histological studies that report a thickening of the retinal layers over time, an optical coherence tomography segmentation technique was used to demonstrate that the combined retinal pigment epithelium and Bruch’s membrane layer thickness linearly decreases with age at the central subfield, inner macula, and outer macula [90]. Macular vascular flow declines with age, which is secondary to a decreased number of vessels and flow velocity [4,91]. Foveal blood flow is diminished in the elderly due to a lower density and diameter of choroidal vasculature supplying the area [88,92].

### 3.5. Optic Nerve

Age-related changes of the optic nerve include swelling of axons at the lamina cribrosa, a reduction in nerve fiber density, and an increase in connective tissue composition such as elastic fibers [4,93,94,95,96]. Furthermore, neural rim volume and minimum rim width of the optic nerve head appear to decline with advancing age [97]. Patients with glaucoma have a significantly higher loss of disc rim compared to non-glaucomatous patients [94]. Older patients have been shown to have lower perfusion of the optic nerve head compared to younger patients [98].

## 4. Age-Related Eye Disease and the Role of Oxidative Stress

### 4.1. Role of Oxidative Stress in Aging

**Figure 4:**
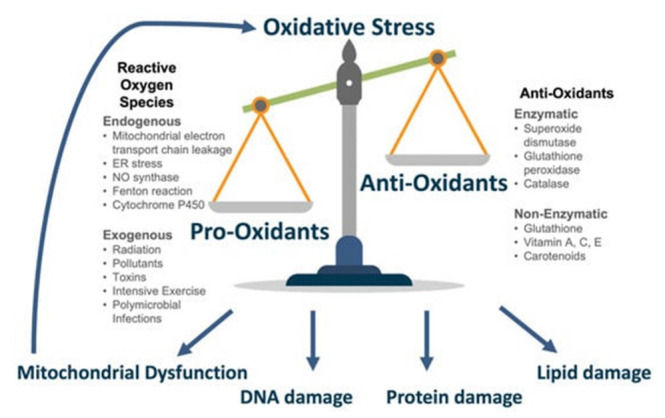
The role of oxidative stress in age-related eye disease [100].

According to the free radical theory of aging, both the natural processes of aging and age-linked diseases are driven by the accumulation of reactive oxygen species (ROS), such as superoxide, hydrogen peroxide, and hydroxyl radicals, from aerobic metabolism [99]. ROS mediate oxidative stress through the chemical modification and damage of connective tissue fibers, DNA, proteins, and lipids [99]. This age-dependent increase in ROS is also accompanied by diminished antioxidant levels, including superoxide dismutase, Vitamin C, and Vitamin E [99]. Oxidative stress plays a significant role in several of the most common age-linked eye diseases (Figure 4) [100].

### 4.2. Cataracts

The 2010 United States prevalence for cataracts was 15.45% in individuals aged 60–64 years and 68.30% for individuals aged 80 years or older [101]. Patients commonly present with a painless progressive decline in visual acuity. Other symptoms, including photophobia, monocular diplopia, myopic shift, and impaired color vision, are based on the type of age-related cataract—nuclear sclerotic, cortical, or posterior subcapsular [102].

Clinical history and ocular examination are used to diagnose cataracts. Nuclear sclerotic cataracts develop as the lens fibers increase in number and become compressed with age. The lens may appear yellow, and the patient may experience a myopic shift, reduced color discrimination, and worsened visual acuity. Wedge-shaped spokes within the peripheral lens cortex are pathognomonic of cortical cataracts, which appear white. Patients with cortical cataracts may present asymptomatically, especially when the central visual axis is spared. In contrast, patients with posterior subcapsular cataracts commonly complain of glare intolerance. On the slit lamp exam, there are granular opacities in the central posterior cortex under the posterior capsule [102].

Oxidative stress underlies the pathogenesis of cataract formation. There is an accumulation of ROS through the upregulation of pro-oxidant enzymes in the lens, such as nicotinamide adenine dinucleotide phosphate oxidase and xanthine oxidase. ROS chemically modify other macromolecules including lipids and proteins. These chemical modifications include oxidation, deamidation, racemization, glycation, methylation, and truncation [52,103,104,105,106,107,108,109]. Affected proteins may form insoluble complexes that contribute to cataractogenesis [52]. ROS also cause DNA damage and lipid peroxidation in lens epithelial cells [109,110]. Additionally, the rise in oxidants leads to the depletion of antioxidants, including reduced glutathione and ascorbate [111]. The Antioxidants for the Prevention of Cataracts (APC) study investigated the impact of antioxidant supplementation with vitamin C, vitamin E, and beta carotene on self-reported cataract extraction. There was no significant difference in the rate of cataract surgery between those receiving vitamin supplementation and those in the placebo group over a 15-year follow-up period [112].

Age-related ion-pump disruption of transmembrane Na^+^/K^+^-ATPase and Ca^2+^-ATPase also contributes to cataract formation by increasing intracellular sodium, calcium, and water content. This leads to lens swelling and opacification [52,113]. The high levels of intracellular calcium promote ROS generation and the release of apoptotic factors leading to cell death [114,115].

### 4.3. Glaucoma

The global prevalence of glaucoma is expected to reach 111.8 million by 2040, with a greater disease burden in Asia and Africa [116]. Primary open-angle glaucoma is common with increasing age [117] and is more prevalent in Black and Hispanic/Latino patients [117,118]. The optic nerve damage of glaucoma may be secondary to ocular hypertension or ischemia of the optic nerve head and, when left untreated, can progress to irreversible loss of peripheral vision and blindness [119]. Diagnosis is made with a battery of visual tests including perimetry, gonioscopy, intraocular pressure testing, optical coherence tomography for evaluation of the retinal nerve fiber layer and ganglion cell thickness, and pachymetry [120].

While the pathology of glaucoma is multifactorial, a diminished response to oxidative stress, mediated by an accumulation of reactive oxygen species, plays a role in disease progression. Enzymatic and nonenzymatic antioxidants counteract ROS. While enzymatic antioxidants, such as superoxide dismutase and glutathione peroxidase, increase in activity with age [121], the levels of nonenzymatic antioxidants, such as vitamin C and vitamin E, decline over time [122,123,124]. There is lowered enzymatic antioxidant activity in the trabecular meshwork and depressed total antioxidant activity in the aqueous humor of patients with glaucoma [125]. As a result, studies have demonstrated elevated levels of oxidative stress markers, including protein carbonyls, advanced glycation end products, malondialdehyde, and DNA damage markers, in aqueous humor samples [125,126,127].

The impaired response to oxidative stress is partially due to mitochondrial dysfunction. The mitochondria of metabolically active tissues, such as the retina and optic nerve, are responsible for energy generation, cell survival and death, and ROS production and consumption [128]. Therefore, mitochondrial DNA mutations and slowed mitophagy lead to age-related increases in ROS production [129,130]. Overall, oxidative stress decreases the number of trabecular meshwork cells and causes aqueous humor outflow resistance to rise, resulting in higher intraocular pressure (IOP) [131]. Elevated IOP causes optic nerve pathology including compression of the lamina cribrosa, obstruction of axoplasmic flow, disruption of retrograde transport to retinal ganglion cells (RGCs), and apoptosis of RGCs [119,131].

Chaudhry et al. reviewed studies investigating nutritional supplementation on glaucoma progression. Supplementation with lutein and zeaxanthin, nitric oxide synthase, crocin, zinc, ginkgo biloba, curcumin, or flavonoids decreased RGC loss or extended RGC lifespan. Additionally, treatment with nicotinamide, flavonoids, resveratrol, crocin, ginkgo biloba, or alpha-tocopherol was found to significantly improve ocular blood flow or protect against ischemia [132].

### 4.4. Age-Related Macular Degeneration

The national prevalence of AMD, based on the 2005–2008 National Health and Nutrition Examination Survey, is 6.5% [133]. It is estimated that the global prevalence of AMD will reach 288 million by 2040 [134]. Risk factors include advancing age, smoking, and hypertension [135]. Early AMD may be asymptomatic, while patients in the late stages of the disease may experience blurry vision, central vision loss, visual distortion, and central scotoma. In addition to clinical history and a fundoscopic examination, diagnosis involves imaging with fluorescein angiography and optical coherence tomography [135].

AMD is classified by early, intermediate, and late stages of disease [136]. Early stages feature drusen deposits. During intermediate stages, these drusen increase in size, number, and pigmentation. Late stages of AMD may be “wet” or “dry”. Wet AMD involves the formation of new abnormal choroidal vessels that are leaky and lead to edema or hemorrhage. Choroidal neovascularization (CNV) is due to the upregulation of angiogenic factors, such as vascular endothelial growth factor (VEGF), after vascular loss and infiltration by macrophages and foreign body giant cells [137]. These vessels may form anastomoses with retinal vessels in advanced stages of neovascular AMD [138]. Intravitreal injections of anti-VEGF medications are the gold standard treatment for CNV in wet AMD. Geographic atrophy is characteristic of late-stage dry AMD [136]. The areas of hypopigmentation seen on fundoscopic exams of patients with geographic atrophy represent degeneration of retinal photoreceptors, RPE, and choriocapillaris [135].

Oxidative stress underlies the pathophysiology of AMD [139]. Harmful reactive oxygen intermediates are produced at the retina from oxygen consumption, irradiation, oxidation of the fatty acids of the photoreceptor outer segment membrane, photosensitizers at the neurosensory retina and the RPE, and RPE phagocytosis [140,141,142]. With advancing age, there is an increased formation of ROS and a decrease in antioxidant levels, resulting in retinal damage.

Several studies have investigated dietary nutrition treatment in AMD. In a follow-up study to the Age-Related Eye Disease Study 2 (AREDS2), there was a significantly lower risk of the development of late AMD in patients receiving supplementation with lutein/zeaxanthin, which are known to defend against ROS at the macula [143,144]. Feng et al. found that dietary lutein improves macular pigment optical density, visual acuity, and contrast sensitivity. The increase in macular pigment optical density appeared to be dose- and treatment duration-dependent [145]. In a randomized controlled trial, the addition of docosahexaenoic acid, lutein, zeaxanthin, resveratrol, and hydroxytyrosol to the original age-related eye disease (ARED) formula (intervention group) reduced inflammatory cytokine levels in patients with unilateral wet AMD compared to subjects receiving the original ARED formulation alone (control group). However, there was no significant improvement in visual acuity after one year between the two groups [146]. Supplementation with oral Macuprev^®^, which includes lutein, zeaxanthin, N-acetylcysteine, as well as vitamins D_3_, B_12_ and C, among other components, was found to increase the amplitude density from multifocal electroretinogram at the central macular area in patients with intermediate AMD, indicating that Macuprev^®^ supplementation may improve macular function [147]. In this disease process, lipoproteins are deposited as drusen at Bruch’s membrane. It has been hypothesized that supplementation with lutein and zeaxanthin modify high- and low-density lipoproteins, which decreases their uptake by RPE uptake receptors, thereby lowering drusen volume at Bruch’s membrane and the risk for neovascularization in AMD [148].

Previous studies have shown an age-dependent accumulation of mitochondrial damage as well as a rise in the levels of lipid peroxidation, advanced glycation end products, lipofuscin, carboxyethylpyrrole proteins, and 8-oxo-7,8-dihydro-2′-deoxyguanosine [149,150,151]. Murine models showed that mice with lower levels of superoxide dismutase, an antioxidant, had higher ROS levels and developed AMD characteristics including drusen, thickened Bruch’s membrane, and CNV [152,153]. ROS also upregulate VEGF levels in the RPE, leading to the CNV seen in wet AMD [154,155]. Cigarette smoking, a high-fat diet, and excess light exposure have been identified as sources of ROS and modifiable environmental risk factors for AMD [154].

Complement system dysfunction also contributes to the pathophysiology of AMD [131]. Intermediate- and late-stage AMD patients were found to have greater levels of complement activation in the intercapillary septa and Bruch’s membrane compared to early-stage and healthy patients [156]. Complement factors C3a and C5a promote mast cell degranulation and the subsequent atrophy of the extracellular matrix seen in geographic atrophy. The atrophy of the extracellular matrix is mediated by released proteinases such as tryptase [136,157]. Other proteolytic enzymes cause choroidal atrophy and thinning as well as the CNV formation seen in wet AMD [136,158]. The deposition of oxidation products, complement proteins, and membrane attack complexes within soft drusen leads to a local inflammatory response [136].

## 5. Conclusions

The structural and functional changes that occur during the natural aging process at both the anterior and posterior segments of the eye lead to several visual deficits among the elderly and affect their activities of daily life as well as their mental health. The oxidative stress driving these changes is also present in age-related ocular pathologies including glaucoma, cataracts, and macular degeneration. Understanding the biochemical processes underlying these age-related changes is key to discovering therapeutic targets that may prevent or slow the progression of these changes in the eye.

## Figures and Tables

**Table 1:** Current and future prevalence of age-related eye diseases in the United States.

Disease	2010	2030	2050
Cataracts	24,409,978	38,737,561	50,231,932
Glaucoma	2,719,379	4,284,823	6,290,760
Age-Related Macular Degeneration	2,069,403	3,664,044	5,442,265

Source: https://www.nei.nih.gov/learn-about-eye-health/eye-health-data-and-statistics (accessed on 7 February 2023).

**Table 2:** Summary of the age-related changes in the anterior segment of the eye that are discussed in the present work.

Anatomical Feature	Age-Related Changes
Eyelids and Lacrimal Glands	Horizontal lid laxity
	Ectropion
	Entropion
	Dermatochalasis
	Blepharoptosis
	Gland atrophy and fibrosis
Sclera	Increase in stiffness and rigidity
	Senile scleral plaques
Cornea	Thickening of the Descemet’s and epithelial basement membranes
	Decrease in corneal stromal density
	Decrease in corneal endothelial cell (Fuchs’ dystrophy) and conjunctival keratocyte numbers
	Shift from with-the-rule astigmatism to against-the-rule astigmatism
	Arcus senilis
	Cornea farinata
	Crocodile shagreen
	Phagocytic dysfunction and loss of phagocytically active cells
Trabecular Meshwork	Reduction in height
	Decrease in cellularity
	Increase in extracellular components
	Hyperpigmentation
Ciliary Body	Shortening and widening of the muscle
	Decrease in diameter
	Loss of vascularization and cellularity
Crystalline Lens	Increase in radii of curvature, lens volume, surface area, cross-sectional area, diameter, lens thickness, and weight
	Blue blindness
	Cataract formation
	Presbyopia secondary to increased stiffness

**Table 3:** Summary of the age-related changes in the posterior segment of the eye that are discussed in the present work.

Anatomical Feature	Age-Related Changes
Vitreous Humor	Aggregation of collagenous fibrils
	Thickening of the vitreous base
	Increased stiffness, dehydration, and mobility
Retina and Retinal Pigment Epithelium	Neuronal cell loss
	Diminished total retinal blood vessel area
	Decrease in the number of pericytes and endothelial cells
	Retinal pigment epithelium cell vacuolization and loss of cytoplasm
	Accumulation of lipofuscin
	Impaired melanin antioxidant properties
Choroid	Lower mean thickness, vessel volume, and stroma volume
	Diminished choriocapillaris density and diameter
	Loss of melanosomes within choroidal melanocytes
Macula and Fovea	Increase in macular retinal pigment epithelium cell height
	Thickening and calcification of Bruch’s membrane
	Drusen deposition
	Decline in macular vascular flow
Optic Nerve	Reduction in nerve fiber density
	Lower neural rim volume and minimum rim width
	Decreased perfusion of optic nerve head

## Data Availability

No new data were created or analyzed in this study. Data sharing is not applicable to this article.
